# Biportal Endoscopic Decompression with Maximized Facet Joint Preservation for Central to Extraforaminal Lumbar Stenosis

**DOI:** 10.3390/jcm14082725

**Published:** 2025-04-15

**Authors:** Sub-Ri Park, Sung-Ryul Choi, Nam-Hoo Kim, Hak-Sun Kim, Ji-Won Kwon, Kyung-Soo Suk, Seong-Hwan Moon, Si-Young Park, Jae-Won Shin, Byung-Ho Lee, Jin-Oh Park

**Affiliations:** 1 Department of Orthopedic Surgery, Yongin Severance Hospital, Yonsei University College of Medicine, Yongin-si 16988, Republic of Korea; ncd1896@yuhs.ac (S.-R.P.);; 2 Department of Orthopedic Surgery, Spine and Spinal Cord Institute, Gangnam Severance Hospital, Yonsei University College of Medicine, Seoul 06273, Republic of Korea; 3 Department of Orthopedic Surgery, Yonsei University College of Medicine, Seoul 03722, Republic of Korea

**Keywords:** biportal endoscopic spine surgery, foraminal stenosis, ipsilateral decompression, contralateral decompression, transforaminal approach, interlaminar approach

## Abstract

**Background/Objectives:** This is a retrospective study. We aimed to identify an optimal biportal endoscopic spine surgery (BESS) technique that maximizes facet joint preservation while achieving sufficient decompression for central to extraforaminal lumbar stenosis across all spinal levels. **Methods:** We retrospectively analyzed the data of 46 patients who underwent surgery and assessed clinical outcomes (visual analogue scale scores for pain; pregabalin usage) and radiological changes (using computed tomography/magnetic resonance imaging) in the spinal canal; intervertebral foramen area expansion; facet joint preservation; and degenerative change. **Results:** Using interlaminar and transforaminal approaches (two-way BESS decompression technique), the mean facet joint volume preservation ratio was 87%, and the mean facet joint length maintenance ratio was 90%, indicating a successful anatomical preservation compared with previous studies. Radiological outcomes revealed effective decompression (178% in the spinal canal; 245% in intervertebral foramen expansion). Additionally, all clinical outcome parameters significantly improved (*p* < 0.001). **Conclusions:** To the best of our knowledge, this study is the first to accurately estimate the degree of facet joint preservation using different methods after endoscopic surgery. The two-way BESS decompression technique maximized facet joint preservation with sufficient decompression and clinically improved central to extraforaminal stenosis across all lumbar levels. Therefore, this technique can sufficiently preserve facet joints to prevent rapid degenerative change after surgery.

## 1. Introduction

Facet joint preservation is crucial in biportal endoscopic decompression for spine surgery, a procedure that addresses various spinal pathologies. The interlaminar and transforaminal approaches represent two distinct surgical techniques, each offering unique advantages for specific pathologies. Both approaches enable sufficient decompression; however, the interlaminar approach is commonly used for pathologies affecting the central and lateral recesses, while the transforaminal approach is preferred for pathologies affecting the extraforaminal region. As spinal pathologies manifest diversely across different regions, optimizing facet joint preservation requires a nuanced understanding of the efficacy of these approaches in specific cases.

Our study aims to explore and compare the facet joint preservation outcomes using both interlaminar and transforaminal approaches in biportal endoscopic spine surgery (BESS) decompression for central to extraforaminal stenosis. Understanding the nuances of preservation techniques is vital for surgical success, as it can enhance patient outcomes and minimize potential complications associated with facet joint deterioration. Therefore, our study seeks to determine the efficacy of these approaches in maximizing facet joint preservation and improving clinical outcomes across all levels of lumbar stenosis.

## 2. Materials and Methods

### 2.1. Patient Selection

**Figure 1:**
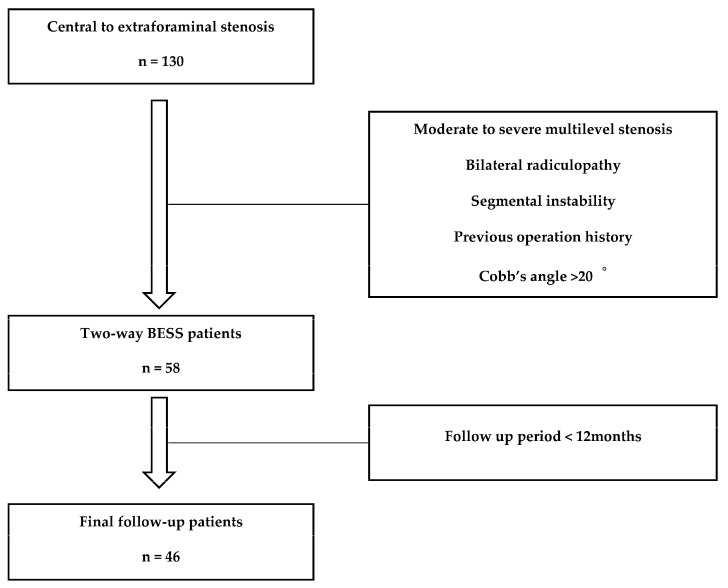
Flowchart of patients included and excluded in this study.

After obtaining approval from the Institutional Review Board, 130 patients with central to extraforaminal stenosis were retrospectively identified between March 2021 and July 2022. All study procedures were performed following relevant guidelines and regulations, with written informed consent obtained from all participants. The follow-up period of these patients was 16.35 ± 2.58 months (mean ± SD). The study was conducted at a single center, and a single surgeon performed the two-way BESS decompression. Clinical signs observed before surgery included lower back pain, intermittent neurogenic claudication, unilateral radiating pain, numbness, and weakness in the lower extremities. To diagnose lumbar spinal stenosis, all patients underwent simple lumbar radiography, computed tomography (CT), and magnetic resonance imaging (MRI). The target patients underwent conservative treatment for more than 6 weeks, including medication, physical therapy, and selective nerve block. The exclusion criteria were (1) patients with segmental instability, (2) degenerative scoliosis Cobb’s angle of >20°, (3) patients with severe complaints of bilateral radiculopathy, (4) patients with a surgical history, (5) moderate to severe multilevel stenosis, and (6) a follow-up period of less than 12 months. After applying all the exclusion criteria, 58 patients underwent two-way BESS decompression. A total of 46 patients were enrolled, excluding those who did not complete a 12-month follow-up period. The overall patient selection flowchart is illustrated in Figure 1.

### 2.2. Surgical Procedure

#### 2.2.1. Interlaminar Approach

During surgeries performed under general or epidural anesthesia, patients were placed in a prone position. Anatomical landmarks, including the surgical site, pedicle, and spinous process, were marked using a C-arm X-ray for precise surgical access. The interlaminar space portals varied by disc and space levels, requiring precise incisions (approximately 1 cm inside the pedicle’s medial border and 1 cm above and below the interlaminar space), and were adjusted for factors such as lordosis or degenerative changes. Radiofrequency or bone wax was used to manage the bleeding. The initial steps involved drilling the ipsilateral lamina and spinous process base to allow instrument movement. A cranial laminotomy was performed to access the ligamentum flavum (LF) notch. The LF was then dissected, ensuring protection of the dural sac. Drilling the contralateral lamina exposed the lateral recess. Careful examination of the dural sac and root route was performed after LF removal. Foraminal decompression was performed, avoiding facet joint violation while removing any bony spur or herniated disc. A 30° endoscope enhanced the visibility of the medial facet joint, examining the superior articular process (SAP) tip and root pathway. The same techniques were applied on the opposite side using tools such as hockey chisels, curettes, and Kerrison punches. This systematic approach addressed anatomical specifics at each level to achieve successful surgical outcomes.

#### 2.2.2. Extraforaminal Approach

**Figure 2:**
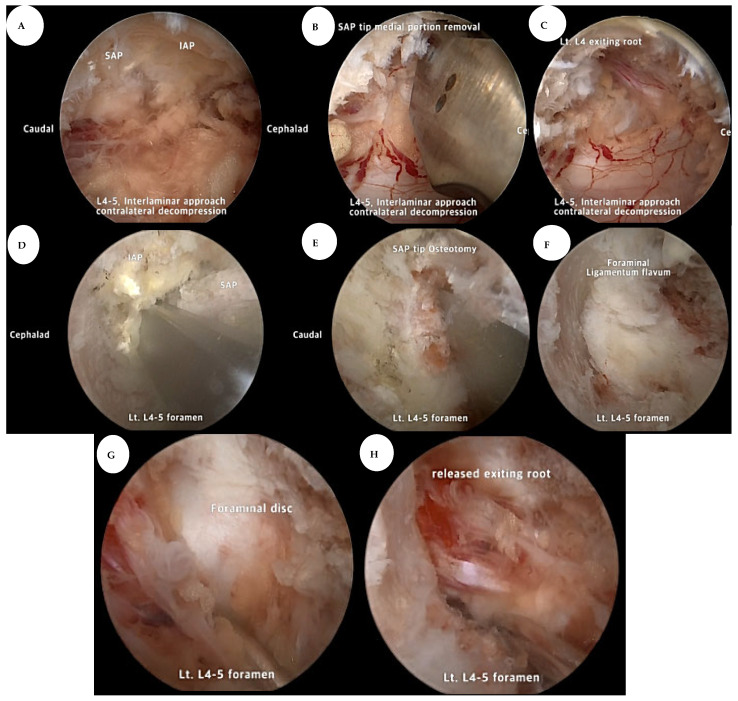
Intraoperative findings. (**A**) Contralateral-side sublaminar decompression with interlaminar approach. We could see sufficient SAP and an inferior articular process medial margin. (**B**) Removal of the medial portion of the SAP tip with an osteotome or Kerrison punch. (**C**) Sufficient decompression of the exiting root pathway in the foramen medial area. (**D**) Ipsilateral transforaminal approach with a 30° endoscope. (**E**) Removal of the lateral portion of the SAP tip with an osteotome or Kerrison punch. (**F**) Removal of the foraminal ligamentum flavum. (**G**) If a foraminal disc was available, sufficient discectomy could be performed. (**H**) Full decompression of the exiting root from the foraminal to the extraforaminal area.

**Figure 3:**
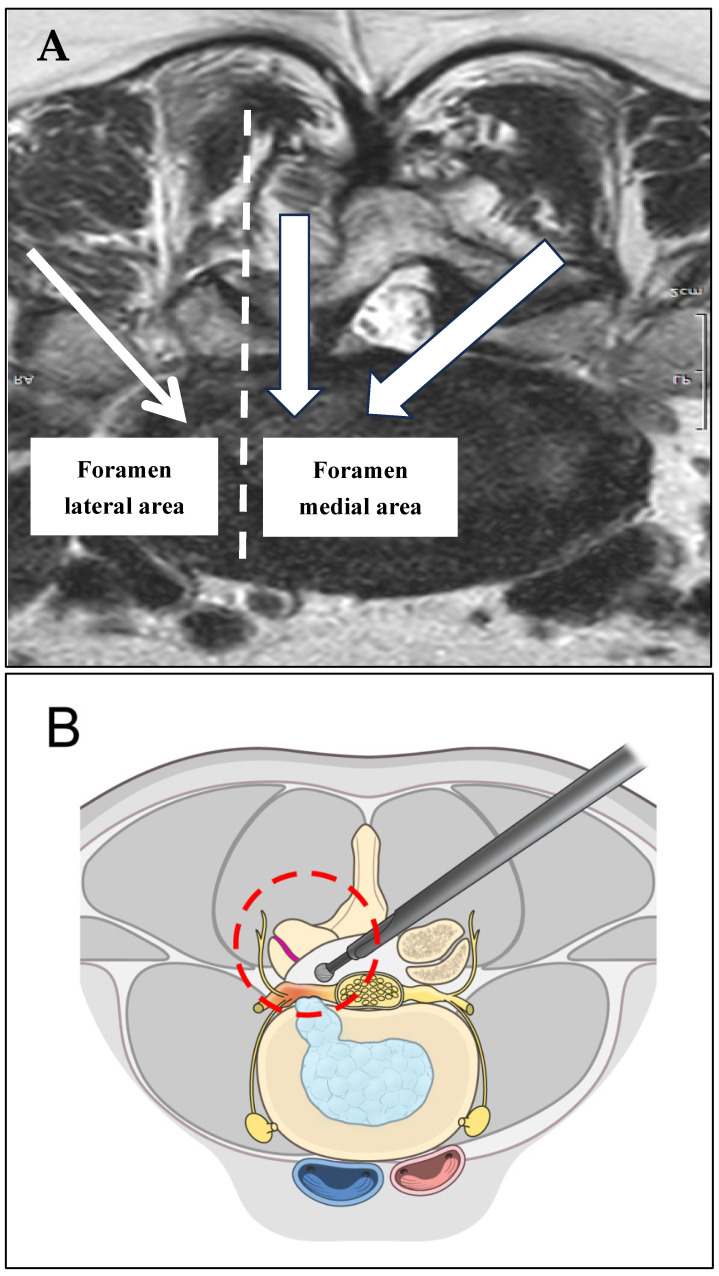

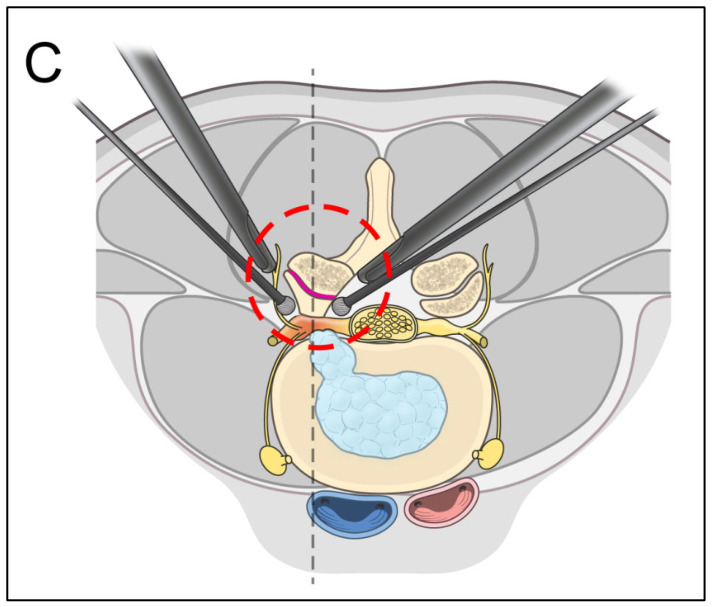
Basic concept of the two-way BESS decompression technique. (**A**) Ipsilateral or contralateral sublaminar decompression with an interlaminar approach can be selected by the surgeon. (Thick arrow) After central to medial foraminal decompression, lateral foraminal and extraforaminal decompression will be performed using an ipsilateral transforaminal approach (thin arrow). (**B**) Using only the contralateral sublaminar endoscopic decompression technique can cause more facet joint damage to enough of the decompression foraminal lateral area and the extraforaminal area; also, insufficient decompression can occur due to various causes. (**C**) Just a little damage to the facet joint from both sides based on the foramen, central to extraforaminal stenosis, can be sufficiently decompressed. The two-way BESS decompression technique can preserve the facet joint as much as possible.

Surgeons utilized anteroposterior C-arm fluoroscopy to pinpoint crucial spinal landmarks for surgery. Strategic vertical incisions were placed about 1 cm from the spinous process at precisely located portals, ensuring optimal visibility. A muscle detacher gently separated soft tissues around the transverse process, facilitating a clear surgical field by allowing examination of fluid outflow and revealing the lateral facet joint capsule. The 30° endoscope enhanced visualization, confirming the foramen location and the tip of the SAP. Specialized tools, such as a diamond burr, pituitary rongeur, and radiofrequency, controlled soft tissue and managed hemorrhage after exposing the isthmus, facet joint, and transverse processes. Precise resection of the SAP tip aided decompression. LF removal, surgical field expansion via Kerrison punches and pituitary rongeurs, and careful decompression in cases of obstructed root pathways were integral. Integrating interlaminar and transforaminal techniques ensured comprehensive decompression across lumbar levels, emphasizing L5-S1, minimizing complications, and prioritizing facet preservation. Figure 2 and Figure 3 illustrate a more detailed explanation of the two-way BESS decompression technique step by step.

### 2.3. Outcome Evaluation

**Figure 4:**
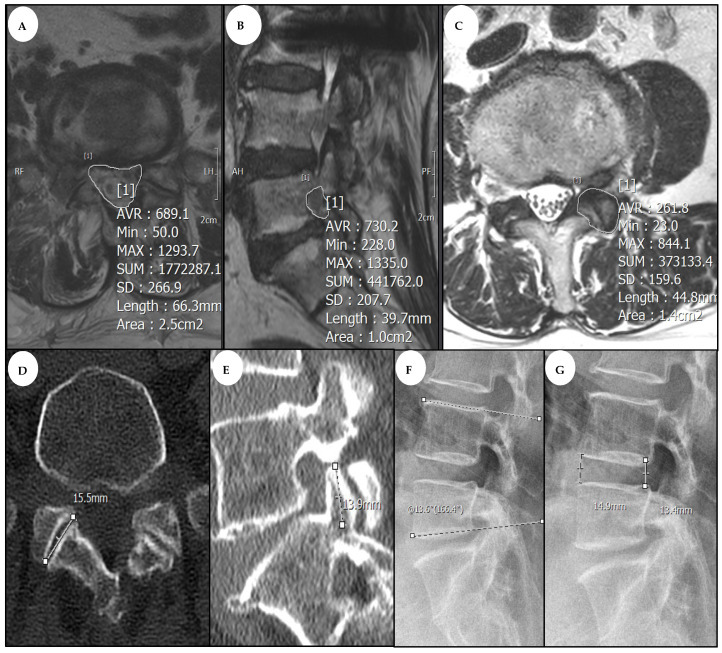
Measurement of radiologic parameters. (**A**) Measurement of CSA-SC in MRI T2 axial view. (**B**) Measurement of CSA-IVF in MRI T2 foraminal sagittal view. (**C**) Measurement of CSA-FJ in MRI T2 axial view. (**D**) Measurement of facet length in CT axial view. (**E**) Measurement of SAP length in CT foraminal sagittal view. (**F**) Measurement of segmental lordosis in lumbar spine lateral plain X-ray. (**G**) Measurement of disc height in lumbar spine lateral plain X-ray.

Clinical outcomes were evaluated using the visual analogue scale (VAS) for back and leg pain and medication (pregabalin) use. Many medications are used to treat spinal stenosis; however, we focused on pregabalin use to assess radiculopathy improvement following surgery. Pre-operative and post-operative evaluations of these parameters were conducted at 1, 3, 6, and 12 months. Additionally, the results of the modified MacNab criteria were assessed pre-operatively and at each follow-up. Radiological outcomes were evaluated using pre-operative and post-operative CT and MRI. The foraminal compression and lateral recess sizes at the same level were assessed on pre-operative CT, and MRI images were analyzed using the ZETTA PACS viewer system (version 2.0.3.6.2.1, Taeyoung Soft Co., Ltd., Gwacheon-si, Republic of Korea). Post-operative CT and MRI were performed 12 months after surgery to assess the effectiveness of decompression. For morphometric analysis, the cross-sectional area (CSA) of the spinal canal (CSA-SC), intervertebral foramen (CSA-IVF), and facet joint (CSA-FJ) at the level of foraminal decompression were measured using a T2-weighted MRI. An arbitrary line was drawn around the region between the facet and the lamina to calculate the CSA-SC. The CSA-IVF was calculated by drawing an imaginary circle around the neural foramen on the side of the parasagittal cuts associated with the symptoms. A hypothetical line was drawn around the facet joint at the site of the impacted foraminal compression to calculate the CSA-FJ. All areas were measured in millimetres. Disc height was calculated using the average value of the height before and after the disc on X-ray, and segmental lordosis was calculated using the degree between the upper vertebral body and the lower vertebral body endplate line. Figure 4 illustrates all these parameters.

### 2.4. Statistical Analyses

To determine the differences between the percentages of CSA-SC and CSA-IVF in the degree of decompression, CSA-FJ, disc height, segmental lordosis for the preservation of the facet joint, and other structures between the pre-operative and post-operative statuses, we first confirmed whether the assumption of normality was satisfied by performing normality tests. For each parameter, a repeated-measures ANOVA was conducted to assess the differences between pre-operative and post-operative values. In addition, the repeated-measures ANOVA was used to check if the sphericity test was met and to identify significant differences in patient symptoms and pregabalin reduction over time based on the *p*-value in the within-subject effect test. Post hoc analysis was conducted to test for differences according to the time point within the group. The significance level was corrected using Bonferroni’s method. All statistical analyses were performed using SAS version 9.4 (SAS Institute Inc., Cary, NC, USA), with the significance level set at *p* < 0.05.

## 3. Results

### 3.1. Patient Demographics

The characteristics and demographic statistics of the patient groups are summarized in Table 1. The study included 12 men and 34 women with a median age of 71.40 years.

### 3.2. Surgical Outcomes

Table 1 summarizes the surgical results and descriptive information comparing the patient groups according to the surgical level. There were six patients in L3-4, eighteen in L4-5, and twenty-two in L5-S1. The average duration was approximately 2.78 days for the patients. All patients were able to walk the day after surgery. The average operation time was approximately 98 min, and the average bleeding loss was approximately 64 cc. The mean follow-up period was 16.3 months. Complications were reported in two cases. One patient complained of mild headaches after surgery but recovered the day after surgery. The other patient, who was obese, developed a small post-operative retroperitoneal ascites. This was likely caused by fluid entering the retroperitoneal space owing to muscle detachment between the transverse processes during the transforaminal approach. The patient fully recovered before discharge without special complications.

### 3.3. Evaluation of Radiological Outcomes

Statistics of the radiological outcomes before and after surgery in the patient group are presented in Table 2 and Table 3. The mean pre-operative and post-operative CSA-SC values were 1.27 ± 0.23 mm^2^ and 2.27 ± 0.27 mm^2^, respectively. The mean pre-operative and post-operative CSA-IVF values were 0.46 ± 0.13 mm^2^ and 1.13 ± 0.16 mm^2^, respectively. The maintenance ratios of CSA-FJ, facet joint length, and SAP length were 87.34%, 90.05%, and 75.55%, respectively. Furthermore, the maintenance ratios of disc height and segmental lordosis were 98.81% and 98.44%.

### 3.4. Clinical Outcomes

The pre-operative back VAS (estimated mean) was 4.74, which improved to 2.44, 2.04, 1.39, and 1.17 at 1 month, 3 months, 6 months, and 1 year post-operatively, respectively (*p* < 0.001). The pre-operative leg VAS (estimated mean) was 7.52, which improved to 3.44, 2.78, 2.17, and 1.96 at 1 month, 3 months, 6 months, and 1 year post-operatively, respectively (*p* < 0.001). The amount of pregabalin administered before surgery was 247.83 mg, which decreased to 102.17, 63.04, 33.70, and 18.48 mg at 1 month, 3 months, 6 months, and 1 year post-operatively, respectively (*p* < 0.001). Table 4 presents the changes in the back VAS, leg VAS, and amount of pregabalin usage during the follow-up period.

The global results based on the modified MacNab criteria were as follows: Pre-operatively, 12 patients (55.2%) had poor results, and 11 (47.8%) had fair results. One month after surgery, 9 patients (39.1%) had fair results, and 14 patients (60.9%) had good results. Three months after surgery, 4 patients (17.4%) had fair results, 16 patients (69.6%) had good results, and 3 patients (13.0%) had excellent results. Six months after surgery, 2 (8.7%) patients had fair results, 16 (69.6%) had good results, and 5 (21.7%) had excellent results. Twelve months after surgery, 9 (39.1%) had good outcomes and 14 (60.9%) had excellent outcomes (Table 5).

### 3.5. Case Report

**Figure 5:**
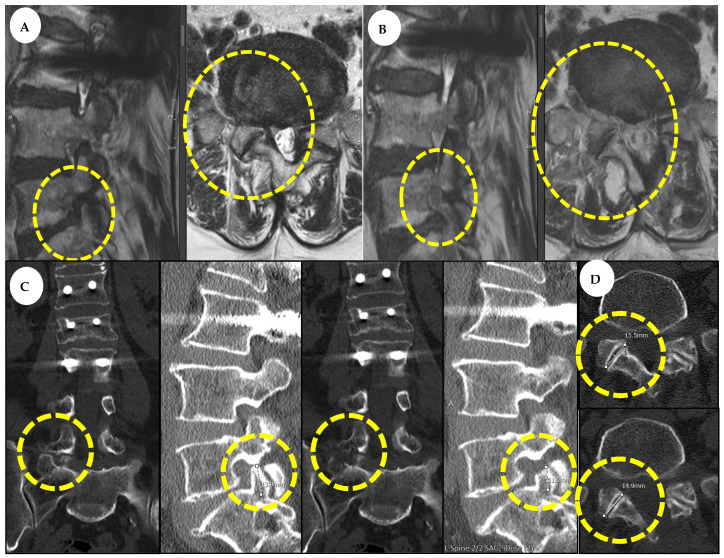
Radiological difference after two-way BESS decompression. The yellow circles indicate the lesion areas where preoperative and postoperative changes can be observed. (**A**) Pre-operative MRI; right-side L5-S1 lateral recess and extraforaminal stenosis were observed. (**B**) Post-operative MRI; sufficient decompression in the right side of L5-S1 was observed. (**C**) Pre-operative and post-operative coronal and sagittal cuts from CT scan; SAP tip was decreased by only 0.6 mm. (**D**) Pre-operative and post-operative axial cut from CT scan; joint length was decreased by only 2.9 mm. MRI, magnetic resonance imaging; CT, computed tomography; SAP, superior articular process.

In a 68-year-old female patient presenting with radiculopathy of the right L5 dermatome, conservative treatment yielded no improvement. The patient exhibited lateral recess and extraforaminal stenosis at the right L5-S1 level on MRI and CT. The back and leg VAS scores were both 7. Consequently, the patient underwent L5-S1 decompression using both interlaminar and transforaminal approaches. Comparing the pre-operative and post-operative assessments, the expansion ratio of the CSA-SC was 184.62%, the CSA-IVF expansion ratio was 240%, and the CSA-FJ preservation ratio was 89.47%. Additionally, post-operative assessments revealed a 0.6 mm decrease in joint length and a 2.9 mm decrease in SAP length compared with pre-operative measurements, indicating successful preservation. The patient was discharged on post-operative day 3 and exhibited significant improvement at the 1-year follow-up, with back and leg VAS scores reduced to 2 each, meeting the MacNab criteria for an excellent outcome. Pre-operative and post-operative CT and MRI scans are illustrated in Figure 5.

## 4. Discussion

Symptomatic lumbar stenosis, from the center to the extraforaminal region, causes severe impairment due to both traversing and exiting nerve root dysfunction [1]. Decompression with interbody fusion surgery is regarded as the gold-standard treatment for these lesions. However, open lumbar surgery can cause complications, such as wound problems, operation-site muscle fibrosis, longer hospitalizations, infections, adjacent segment disease, instrumental failures, and pseudoarthrosis. Numerous minimally invasive non-fusion procedures have been developed to address these issues, with some reports favouring the selective application of endoscopic decompression to areas requiring direct decompression following indirect decompression [2]. 

Many studies have reported numerous advantages of endoscopic spine surgery; however, facet joint preservation is the most crucial consideration in non-fusion spinal surgery [3,4,5,6,7]. Wangsawatwong et al. demonstrated that when a facet violation occurs, the mobility of the superior adjacent level increases, and the strains of the disc surface change, accelerating degenerative changes. Therefore, facet preservation is necessary to prevent degeneration [8,9,10]. Kim et al. demonstrated that more than 50% of facet joint preservation can be achieved when BESS is performed in patients with lumbar spinal stenosis. Young et al. were the first to compare the degree of facet joint preservation between the ipsilateral and contralateral approaches in biportal endoscopic decompression [11]. They observed better preservation of facet length in the contralateral approach than in the ipsilateral approach (91.9% vs. 83.7%) [12]. Despite BESS leading to fewer iatrogenic injuries due to its flexible manipulation and good visualization, challenges remain in overcoming facet joint violations in ipsilateral approach surgery [11]. Song et al. reported that the violation of the medial facet joint is inevitable for adequate surgical field exposure in the ipsilateral approach, especially in conditions such as facet hypertrophy combined with foraminal stenosis [13]. Kim et al. also reported that ipsilateral facet violation is unavoidable in achieving sufficient decompression through either the interlaminar or transforaminal approach alone [14]. To overcome these challenges, many decompression studies using various techniques are in progress and have been reported. However, limitations persist when only the ipsilateral or transforaminal approach is used.

Kim et al. demonstrated both good clinical outcomes and facet preservation in complete endoscopic contralateral foraminal and lateral recess decompression in unilateral foraminal stenosis [15]. However, they did not provide exact values for the extent of facet joint preservation, and various instruments were required for the next steps. In another study, Kim et al. reported that contralateral sublaminar decompression using biportal endoscopy for lateral recess to extraforaminal stenosis might result in insufficient decompression of the extraforaminal area, necessitating additional decompression with a paraspinal approach in such cases [16]. 

The focus of our study was to achieve sufficient decompression efficiently while preserving the facet joint as much as possible at all lumbar levels. We hypothesized that combining existing approaches in BESS techniques would be more advantageous than using only one approach, similar to knee or shoulder arthroscopic surgery, which utilizes various portals. Therefore, we used two existing approach methods: the interlaminar and transforaminal approaches simultaneously. The central canal and the medial foramen area were decompressed using the interlaminar approach, while the lateral foramen to the extraforaminal area was decompressed with the transforaminal approach. We termed this method “two-way BESS decompression”. Our study revealed the following advantages.

First, combining these approaches allows us to address various issues easily. An ipsilateral- or contralateral-side approach may be required depending on the facet shape abnormality, degenerative lumbar scoliosis, hypertrophic bony structures, or the location of the disc material. In these cases, the ipsilateral- or contralateral-side lesion from the canal to the foramen medial area is decompressed through the ipsilateral approach, minimizing facet joint violation. The remaining lesion is decompressed through the transforaminal approach, ensuring sufficient decompression regardless of structural changes or the location of the lesion while preserving the facet joint as much as possible.

Second, this method can be applied at all lumbar levels. The L5-S1 level is particularly problematic owing to anatomical factors such as a high iliac crest, hypertrophy of the L5 transverse process, hyperplasia of the articular process, an enlarged lumbosacral angle, a high sacral flank, and a narrow intervertebral foramen. These factors make it challenging to perform sufficient decompression to the central area through the transforaminal approach [16,17]. Additionally, decompression can be challenging with the contralateral interlaminar approach due to the impingement of the L5 nerve root, known as “far-out syndrome.” However, in our study, we demonstrated that the two-way BESS decompression method can be easily and sufficiently used for the decompression of all stenotic lesions at the L5-S1 level.

Third, the facet joint can be sufficiently preserved. Eun et al. reported that when interlaminar BESS decompression was performed in multilevel lumbar stenosis, the ipsilateral facet joint volume was preserved at 91.2%, and the contralateral facet joint volume was preserved at 93.4% [18]. Similarly, Yeung et al. reported that the facet joint length was preserved at 83.7% through the interlaminar ipsilateral approach and 91.9% through the contralateral approach [11]. Tian et al. also reported an average facet joint volume preservation of 89.2% through the contralateral approach [7]. Our study did not reach a consensus on the exact value of facet joint preservation; we measured it using various techniques. We observed that 90.1% of the facet joint length and 87.3% of the facet joint volume were preserved, with approximately 75.6% of the SAP length remaining. Compared with previous results, our findings confirm that the facet joint can be sufficiently preserved even when using two approaches.

Fourth, this method proved efficient when comparing operation time, blood loss, and clinical outcomes. According to Eun et al., when a two-level interlaminar BESS decompression was performed, the mean operation time was 111 min, with a mean blood loss of 88 cc [18]. Tian et al. reported an average operation time of 85 min for contralateral-side interlaminar biportal decompression from the lateral recess area to the foraminal area at the same level [7]. Despite combining the two approaches, the two-way BESS decompression technique was performed in an average of 98 min, with a mean blood loss of about 64 cc. Furthermore, all patients were able to move the next day after surgery; the average hospital stay was 2.78 days, and the average spinal canal and foraminal area expansion was also effective at 178% and 245%, respectively. Clinical symptoms also exhibited sufficient statistical improvement during the one-year follow-up after surgery. Overall, this surgical technique was confirmed to be sufficiently effective.

Finally, in our study, we could not determine the possibility of re-stenosis due to progressing degenerative changes during the average follow-up of 16 months. The heights of the posterior intervertebral disc can affect clinical outcomes because disc space narrowing is likely to cause root compression. Additionally, lumbar lordosis reflects the local alignment of the lumbar spine, and lumbar lordosis and surgical outcomes are significantly correlated in spinal stenosis [19]. We compared the change in disc height and segmental lordosis of the surgical level between pre-surgery and 12 months after surgery. Disc height was maintained at 98.8%, and the segmental lordosis was maintained at 98.4%, confirming that significant degenerative changes did not progress during the follow-up period.

Our study has certain limitations that warrant further investigation. First, the study design involved a single-center retrospective analysis, possibly introducing selection biases such as body mass index, smoking status, and medical comorbidities. Future prospective multicenter studies with larger sample sizes are required to validate our findings. Additionally, long-term follow-up is essential to assess the durability of clinical outcomes and to evaluate the prevention of segmental degeneration. Moreover, as this study targeted patients with central to extraforaminal stenosis and unilateral radiculopathy, additional research is needed to determine whether biportal endoscopic decompression alone can achieve sufficient results for both foraminal stenosis. Finally, this study did not distinguish between patients who underwent discectomy and those who did not. Based on this distinction, we presume that symptom improvement and progression may differ following surgery. Therefore, further studies are required to evaluate disc height and segmental lordosis.

## 5. Conclusions

In patients with central to extraforaminal stenosis, the two-way BESS decompression technique is appropriate for maximizing facet preservation and performing complete central to extraforaminal decompression. Furthermore, this technique exhibits favourable attributes, making it applicable to all lumbar levels. Advantages include reduced bleeding, shorter hospitalization periods, and noteworthy positive clinical outcomes.

## Figures and Tables

**Table 1:** Patient demographics and surgical outcomes.

	Mean ± SD
Age	71.40 ± 6.90
Sex	
F	34 (73.91%)
M	12 (26.06%)
Surgical level	
L3-4	6 (13.04%)
L4-5	18 (39.13%)
L5-S1	22 (47.83%)
Operation time (min)	98.04 ± 10.08
Estimated blood loss (mL)	64.13 ± 10.47
HOD (day)	2.78 ± 0.64
Follow-up period (month)	16.35 ± 2.58

F, female; M, male; SD, standard deviation; HOD, hospital day.

**Table 2:** Radiologic outcomes.

	Pre-op	Post-op	Expansion Ratio (%)	*p*-Value
	Mean ± SD	Mean ± SD		
CSA-SC (mm^2^)	1.27 ± 0.23	2.27 ± 0.27	178.74%	<0.0001
CSA-IVF (mm^2^)	0.46 ± 0.13	1.13 ± 0.16	245.65%	<0.0001

CSA-SC, cross-sectional area of the spinal canal; CSA-IVF, intervertebral foramen; SD, standard deviation.

**Table 3:** Maintenance ratio of radiological parameters.

	Pre-op	Post-op	Maintenance Ratio (%)
	Mean ± SD	Mean ± SD	
CSA-FJ (mm^2^)	2.37 ± 0.44	2.07 ± 0.46	87.34%
Facet joint length (mm)	13.57 ± 1.34	12.22 ± 1.48	90.05%
SAP length (mm)	11.74 ± 1.14	8.87 ± 1.25	75.55%
Disc height (mm)	8.39 ± 1.24	8.29 ± 1.24	98.81%
Segmental lordosis (°)	11.52 ± 3.76	11.34 ± 3.70	98.44%

**Table 4:** Clinical outcomes.

	Pre-op	1 Month	3 Months	6 Months	12 Months	Overall *p*-Value
Back VAS Estimated mean (SE)	4.74 (0.24)	2.44 (0.20)	2.04 (0.17)	1.39 (0.14)	1.17 (0.15)	<0.0001
Leg VAS Estimated mean (SE)	7.52 (0.18)	3.44 (0.16)	2.78 (0.19)	2.17 (0.15)	1.96 (0.15)	<0.0001
Pregabalin (mg) Estimated mean (SE)	247.83 (15.23)	102.17 (7.36)	63.04 (8.30)	33.70 (5.80)	18.48 (4.22)	<0.0001

VAS, visual analogue scale; SE, standard error.

**Table 5:** Modified Macnab criteria.

Grade	Pre-op		1 Month		3 Months		6 Months		12 Months	
Poor	12	52.2%	-	0%	-	0%	-	0%	-	0%
Fair	11	47.8%	9	39.1%	4	17.4%	2	8.7%	-	0%
Good	-	0%	14	60.9%	16	69.6%	16	69.6%	9	39.1%
Excellent	-	0%	-	0%	3	13.0%	5	21.7%	14	60.9%

## Data Availability

Data reported were obtained from papers included in the references.
